# Patient–provider discussion about emotional and social needs, mental health outcomes, and benefit finding among U.S. Adults living with cancer

**DOI:** 10.1002/cam4.3918

**Published:** 2021-05-07

**Authors:** Young‐Rock Hong, Sandhya Yadav, Ryan Suk, Ahmad Khanijahani, Daniel Erim, Kea Turner

**Affiliations:** ^1^ Department of Health Services Research Management and Policy College of Public Health and Health Professions University of Florida Gainesville FL USA; ^2^ UF Health Cancer Center Gainesville FL USA; ^3^ Center for Health Services Research Department of Management, Policy and Community Health The University of Texas Health Science Center School of Public Health Houston TX USA; ^4^ Rangos School of Health Sciences Duquesne University Pittsburgh PA USA; ^5^ Parexel International Durham NC USA; ^6^ Department of Health Outcomes and Behavior Moffitt Cancer Center Tampa FL USA; ^7^ Department of Oncological Sciences University of South Florida Morsani College of Medicine Tampa FL USA

## Abstract

**Background:**

A discussion about patient's nonmedical needs during treatment is considered a crucial component of high‐quality patient–provider communication. We examined whether having a patient–provider discussion about cancer patients’ emotional and social needs is associated with their psychological well‐being.

**Methods:**

Using the 2016–2017 Medical Expenditure Panel Survey‐Experiences with Cancer Survivorship Supplement (MEPS–ECSS) data, we identified the cancer survivors in the United States (US) who reported having a detailed discussion about emotional and social needs during cancer care. We used multivariable logistic regression to assess the association between having a patient–provider discussion and the patients’ psychological well‐being outcomes (depressive symptoms, severe psychological distress, and worrying about cancer recurrence/worsening condition) and benefit finding experience after a cancer diagnosis.

**Results:**

Among 1433 respondents (equivalent to 13.8 million cancer survivors in the US), only 33.6% reported having a detailed patient–provider discussion about their emotional and social needs. Having a discussion was associated with 55% lower odds (odds ratio [OR], 0.45; 95% confidence interval [CI], 0.26–0.77) of having depressive symptoms and 97% higher odds (OR, 1.97; 95% CI, 1.46–2.66) of having benefit finding experience. There was no statistically significant association between patient–provider discussion and psychological distress or worrying about cancer recurrence/worsening.

**Conclusion:**

Detailed patient–provider discussion about the cancer patients’ emotional and social needs was associated with a lower likelihood of depressive symptoms and a higher likelihood of experiencing benefit finding. These findings stress the importance of improving the patient–provider discussion about psychosocial needs in cancer survivorship.

## Graphical abstract

Effective patient–provider communication is an integral part of delivering high‐quality cancer care. Study findings suggest that patient–provider discussions about emotional and social needs have a positive impact on mental health outcomes and attitudes toward cancer.

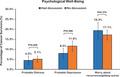

## 1 INTRODUCTION

A cancer diagnosis leads to a number of stressors, such as fear of death, family strain, and financial concerns, that span the cancer care continuum.^1^, ^2^, ^3^, ^4^ Thus, effective and supportive patient–provider communication is required for patients and their families to help meet their physical, emotional, social, psychological, and informational needs during cancer treatment.^5^, ^6^, ^7^, ^8^ However, patients often find it difficult to talk about their worries and other social needs due to fear of discomfort, embarrassment, or disappointment (e.g., provider's unwillingness to address patients’ concerns).^9^, ^10^ Providers may also lack the skills or time necessary to address patients’ emotional or social concerns and tend to avoid discussing patients’ emotional and social issues when they feel incapable of addressing them.^10^ Consequently, many cancer survivors—ranging from 22% to 58% depending on cancer type—experience psychological impairments such as depression, anxiety, and loneliness.^11^, ^12^, ^13^ It is well‐established that psychological distress and depression can have devastating outcomes on cancer survivors, such as worse health‐related quality of life (HRQoL) and a higher risk of cancer‐related mortality or suicide.^14^, ^15^, ^16^, ^17^

Effective patient–provider communication is an integral part of delivering high‐quality cancer care, which helps build a therapeutic relationship between patients and providers.^5^ High‐quality patient–provider communication can improve patient engagement in clinical decision‐making,^18^, ^19^ adherence to medical recommendations,^20^ higher patient satisfaction,^21^, ^22^, ^23^ and resilience in cancer survivorship.^6^ Studies have demonstrated that overall satisfaction with patient–provider communication is associated with better HRQoL and reduced healthcare expenditures.^23^, ^24^, ^25^, ^26^ For example, a recent study of 4,588 cancer survivors found that higher patient satisfaction was associated with higher SF‐12 physical and mental component scores (14%–18% higher) and lower total healthcare expenditures (up to 20% lower in individuals aged >64 years) due to lower rates of hospital admissions and ED visits.^23^

Understanding a patient's psychosocial context, such as their emotional or non‐medical needs, is a critical component of high‐quality, patient–provider communication.^27^, ^28^, ^29^ To date, however, there are limited studies that quantitatively examine what elements of patient–provider communication lead to improved health outcomes, especially mental health outcomes (e.g., distress and resilience). To address this gap, we examine whether having a patient–provider discussion about patients’ emotional and social needs is associated with measures of psychological well‐being among cancer patients. Given the relationship between psychosocial care and benefit finding (e.g., adaptive coping, optimism, resilience),^17^, ^27^, ^28^, ^29^ we also assess the association of having such communication with patients’ attitudes toward living with cancer as a secondary outcome. Evidence of effective communication could help guide efforts to improve patient–provider relationships, development of coping strategies, and quality of life during the course of cancer treatment.

## 2 METHODS

### 2.1 Data and study population

We analyzed the 2016–2017 Medical Expenditure Panel Survey‐Experiences with Cancer Survivorship Supplement (MEPS‐ECSS) data, a nationally representative survey of a non institutionalized U.S. population with a history of cancer that collects information on the burden of cancer care and its impact on health care utilization and expenditure.^30^ The MEPS‐ECSS was administered to a randomly selected subsample of the households responding to the 2015–2016 National Health Interview Survey with a response rate of 81.8% (overall MEPS response rate was 45.1% in 2016–2017).^31^

The study population included 1929 individuals aged 18 years or older who had a confirmed cancer diagnosis. We excluded individuals diagnosed with non‐melanoma or unknown type of skin cancer (n = 404), given its minimal impact on cancer survivorship, consistent with previous work on cancer burden.^32^, ^33^ We also excluded those with unknown history of cancer treatment or being never treated (n = 92) to avoid misclassification errors for patient–provider discussion during cancer care. The final analytic sample consisted of 1433 cancer survivors. This study was deemed exempt from review by the University of Florida Institutional Review Board because we used deidentified, publicly available data. We followed the STROBE (Strengthening the Reporting of Observational Studies in Epidemiology) reporting guidelines.^34^

### 2.2 Exposure: patient–provider discussion about emotional and social needs

Patient–provider discussion about emotional and social needs during cancer care was measured by a question: *“At any time since you were first diagnosed with cancer*, *did any doctor or other healthcare provider*, *discuss your emotional or social needs related to your cancer*, *its treatment*, *or the lasting effects of that treatment?”* Possible response options included: (1) “*discussed it with me in detail*,” (2) *“briefly discussed it with me*,*”* (3) *“did not discussed it at all*,*”* and (4) *“I don't* *remember*.*”* We classified respondents into the discussion or non‐discussion group based on whether they reportedly had indicated patient–provider discussions (set to 1 and 0, respectively). Considering complexities in effective relationship building between patients and providers,^7^, ^8^, ^35^, ^36^ we assumed that patients derived little or no benefit from patient–provider conversations that were short or forgotten. Thus, we excluded individuals that reported brief patient–provider discussions from our main analysis and included these individuals in our sensitivity analyses to examine whether defining discussion differently (e.g., brief discussion included in the definition of discussion) affected the results.

### 2.3 Outcome measures

Outcome measures included indicators of psychological well‐being (i.e., depressive symptoms, severe psychological distress, and worrying about cancer recurrence/worsening) and benefit finding experience (e.g., positive attitudes towards living with cancer).

#### 2.3.1 Depressive symptoms

Depressive symptoms were assessed using the 2‐item Patient Health Questionnaire (PHQ‐2), which has shown to be sensitive in detecting and monitoring depression symptoms. The PHQ‐2 scores range from 0 to 6, and having probable depression was defined if the score was 3 or greater (sensitivity of 83% and specificity of 92% for identifying major depression).^37^ The internal consistency of the PHQ‐2 items in this study was good (Cronbach's alpha = 0.833).

#### 2.3.2 Severe psychological distress

Psychological distress was assessed using the Kessler Index (K6), which is a widely used measure to screen for psychological distress, including nervousness, hopelessness, fidgetiness, sadness, effort, and worthlessness. The K6 score ranges from 0 to 24, and having severe psychological distress was defined as having a score of 13 or higher (sensitivity of 83% and specificity of 92% for identifying severe mental distress).^38^ The internal consistency of the K6 items was excellent (Cronbach's alpha = 0.953).

#### 2.3.3 *Worrying about cancer recurrence*/*worsening condition*

Self‐reported concern related to cancer recurrence or worsening was assessed with a question *“How often do you worry that your cancer may come back or get worse?”* Response scales ranged from 0 *(never)* to *5 (all the time)*. Worrying about cancer recurrence/worsening condition was determined if responses were “*often*” or “*all the time*.”^33^, ^39^

#### 2.3.4 Benefit findings

Four binary questions were used to assess participants’ attitudes toward living with cancer:

1. It has made me a stronger person.
2. I can cope better with life's challenges.
3. It became a reason to make positive changes in my life.
4. It has made me have healthier habits.

We used these four items to create one summary item measuring patients’ benefit findings. Participants reporting at least two “*yes*” responses for any of the four items were categorized as having benefit finding experience. The internal consistency of the four items was good (Cronbach's alpha = 0.820).

### 2.4 Other covariates

As for covariates, we included sample characteristics including quartile age groups (18–54, 55–64, 65–74, 75+), sex, race/ethnicity (non‐Hispanic White, non‐Hispanic Black, Hispanic, Other race), education (less than high school, high school graduate, some college or higher), family income (low income [<200% of federal poverty level (FPL)], middle income [200%‐400% FPL], and high income [>400% FPL]), employment, marital status, census region (Northeast, Midwest, South, and West), type of health insurance (any private, any public, and uninsured), the number of comorbid conditions (hypertension, hyperlipidemia, diabetes, heart diseases, asthma, emphysema, and chronic obstructive pulmonary disease), cancer site (breast, prostate, melanoma, colon, cervical, uterus, lung, lymphoma, bladder, other, and multiple sites), and time since last cancer treatment (on current treatment, 1–4 years, 5–10 years, >10 years).

### 2.5 Statistical analysis

We used descriptive statistics to characterize the study population by the exposure status (patient–provider discussion) using Wald Chi‐square tests. We then examined the association between having patient–provider discussions about emotional and social needs and the outcome measures described above (i.e., depressive symptoms, severe psychological distress, worrying about cancer recurrence/worsening condition, and benefit finding experience) by fitting multivariable logistic regression models. The initial covariate selection was based on previous studies on mental health outcomes and HRQoL among cancer survivors^40^, ^41^ and included in the adjusted analyses based on statistical significance observed (*p* < 0.25) in bivariate analyses.

We also tested the robustness of the main findings by differing the definition of the patient–provider discussion: (1) excluding those who reported “*I don't* *remember*” for whether they had the discussion from the study sample, (2) including only those who reported having a brief patient–provider discussion, and (3) including both detailed and brief discussions combined in the discussion group. We repeated the analyses to assess whether the results differ when including (4) those with non‐melanoma or unknown skin cancer type, (5) those on current treatment only, and (6) those with post‐treatment status only. Recommended survey weight, stratum, and cluster variables were used to account for the complex MEPS survey design and produce nationally representative estimates. Two‐tailed *p* < 0.05 was considered statistically significant. All analyses were performed using SAS statistical software (version 9.4; SAS Institute) from January to July 2020.

## 3 RESULTS

### 3.1 Sample characteristics

Of the 1433 individuals studied (equivalent to a weighted population estimate of 13.8 million cancer survivors in the United States), 33.6% reported having detailed discussions about their emotional and social needs with the provider during cancer care. Table 1 summarizes the characteristics of the study population stratified by whether they had detailed patient–provider discussion about emotional and social needs. Cancer survivors who reported having the discussion were more likely to be younger (34.6% aged under 75 vs. 24.9% aged 75+), racial/ethnic minorities (49.0% non‐Hispanic Blacks vs. 28.7% non‐Hispanic Whites), or married (35.0% married vs. 27.7% not married). By cancer type, those with bladder cancer (41.0%), prostate (39.8%), or breast (36.7%) had a higher prevalence of having the patient–provider discussion.

**TABLE 1:** Characteristics of cancer survivors by patient–provider discussion about emotional and social needs

		Patient–Provider Discussion		
	Total Sample	No	Yes	
	Sample n = 1433	Sample n = 951	Sample n = 482	
	Population Estimate = 13 771 408	Population Estimate = 9 382 324	Population Estimate = 4 389 085	
	**No. (Weighted %)** ^a^	**Weighted Row % (95% CI)** ^a^	**Weighted Row % (95% CI)** ^a^	***p*‐value**
Age group				**0.0130**
18–54	291 (20.2)	65.3 (58.6–72.0)	34.7 (28.0–41.4)	
55–64	306 (21.8)	63.4 (56.4–70.4)	36.6 (29.6–43.6)	
65–74	428 (29.7)	66.9 (61.8–72.0)	33.1 (28.0–38.2)	
75+	408 (28.2)	75.1 (70.2–80.0)	24.9 (20.0–29.8)	
Sex				**0.5771**
Male	569 (40.8)	67.1 (62.1–72.1)	32.9 (27.9–37.9)	
Female	864 (59.2)	68.8 (65.1–72.5)	31.2 (27.5–34.9)	
Race/ethnicity				**0.0003**
Non‐Hispanic White	1009 (80.4)	71.3 (67.8–74.7)	28.7 (25.3–32.2)	
Non‐Hispanic Black	182 (7.4)	51.0 (42.3–59.7)	49.0 (40.3–57.7)	
Hispanic	172 (7.3)	61.2 (51.6–70.9)	38.8 (29.1–48.4)	
Other	70 (5.0)	52.6 (38.0–67.3)	47.4 (32.7–62.0)	
Marital status				**0.0116**
Not married	669 (42.8)	72.3 (68.3–76.3)	27.7 (23.7–31.7)	
Married	764 (57.2)	65.0 (60.9–69.1)	35.0 (30.9–39.1)	
Education				**0.5328**
Less than high school graduate	271 (13.9)	67.0 (60.8–73.3)	33.0 (26.7–39.2)	
High school graduate	595 (41.1)	69.9 (65.4–74.4)	30.1 (25.6–34.6)	
Some college or more	567 (44.9)	66.8 (62.3–71.3)	33.2 (28.7–37.7)	
Family income				**0.0693**
Low income (FPL <200%)	515 (27.2)	62.9 (57.4–68.3)	37.1 (31.7–42.6)	
Middle income (FPL 200–400%)	336 (23.1)	70.7 (65.3–76.0)	29.3 (24.0–34.7)	
High Income (FPL >400%)	582 (49.7)	69.8 (65.4–74.2)	30.2 (25.8–34.6)	
Employment				**0.1281**
Not employed	954 (63.2)	69.9 (66.4–73.3)	30.1 (26.7–33.6)	
Employed	479 (36.8)	65.2 (59.9–70.4)	34.8 (29.6–40.1)	
Region				**0.3112**
Northeast	278 (18.9)	70.4 (62.0–78.8)	29.6 (21.2–38.0)	
Midwest	320 (23.6)	68.8 (62.9–74.7)	31.2 (25.3–37.1)	
South	549 (37.7)	64.8 (60.6–69.1)	35.2 (30.9–39.4)	
West	286 (19.7)	71.5 (65.0–78.0)	28.5 (22.0–35.0)	
Current health insurance				**0.3317**
Private	837 (64.1)	69.3 (65.6–73.0)	30.7 (27.0–34.4)	
Public	566 (33.9)	66.7 (61.7–71.7)	33.3 (28.3–38.3)	
Uninsured	30 (2)	54.1 (30.6–77.6)	45.9 (22.4–69.4)	
Number of chronic conditions^b^				**0.4091**
0	263 (19.4)	70.7 (63.8–77.5)	29.3 (22.5–36.2)	
1	342 (23.5)	71.3 (66.0–76.6)	28.7 (23.4–34.0)	
2	308 (22.6)	65.4 (59.3–71.5)	34.6 (28.5–40.7)	
3+	520 (34.5)	66.3 (60.7–71.9)	33.7 (28.1–39.3)	
Time since last cancer treatment				**0.7429**
On treatment	477 (31.5)	66.1 (61.3–71.0)	33.9 (29.0–38.7)	
Post‐treatment 1 to <5 years ago	284 (20.4)	69.7 (63.2–76.2)	30.3 (23.8–36.8)	
5 to <10 years ago	247 (17.5)	67.0 (60.0–74.1)	33.0 (25.9–40.0)	
10+ years ago	425 (30.6)	69.8 (64.4–75.1)	30.2 (24.9–35.6)	
Cancer site				**<0.0001**
Breast	352 (25.6)	63.3 (57.7–68.9)	36.7 (31.1–42.3)	
Prostate	200 (13.6)	60.2 (53.6–66.8)	39.8 (33.2–46.4)	
Melanoma	94 (7.4)	87.4 (81.4–93.5)	12.6 (6.5–18.6)	
Colon	98 (6)	66.9 (55.4–78.4)	33.1 (21.6–44.6)	
Cervical	88 (5.7)	74.3 (63.9–84.8)	25.7 (15.2–36.1)	
Uterus	63 (3.8)	82.3 (72.2–92.4)	17.7 (7.6–27.8)	
Lung	47 (3.6)	71.3 (57.7–85.0)	28.7 (15.0–42.3)	
Lymphoma	44 (3.1)	64.0 (47.8–80.1)	36.0 (19.9–52.2)	
Bladder	39 (3.4)	59.0 (40.4–77.7)	41.0 (22.3–59.6)	
Other^c^	279 (19.1)	68.9 (62.4–75.3)	31.1 (24.7–37.6)	
Multiple sites	129 (8.7)	71.0 (62.4–79.5)	29.0 (20.5–37.6)	

Abbreviation: FPL, federal poverty level

^a^ Estimates are weighted to be nationally representative using recommended weighting, stratification, and clustering by the Agency for Healthcare Research and Quality

^b^ Including hypertension, hyperlipidemia, diabetes, heart diseases, asthma, emphysema, and chronic obstructive pulmonary disease

^c^ Including bone, brain, esophagus, gallbladder, kidney, larynx, liver, mouth, pancreas, stomach, throat, and thyroid.

### 3.2 Psychological well‐being

**FIGURE 1:**
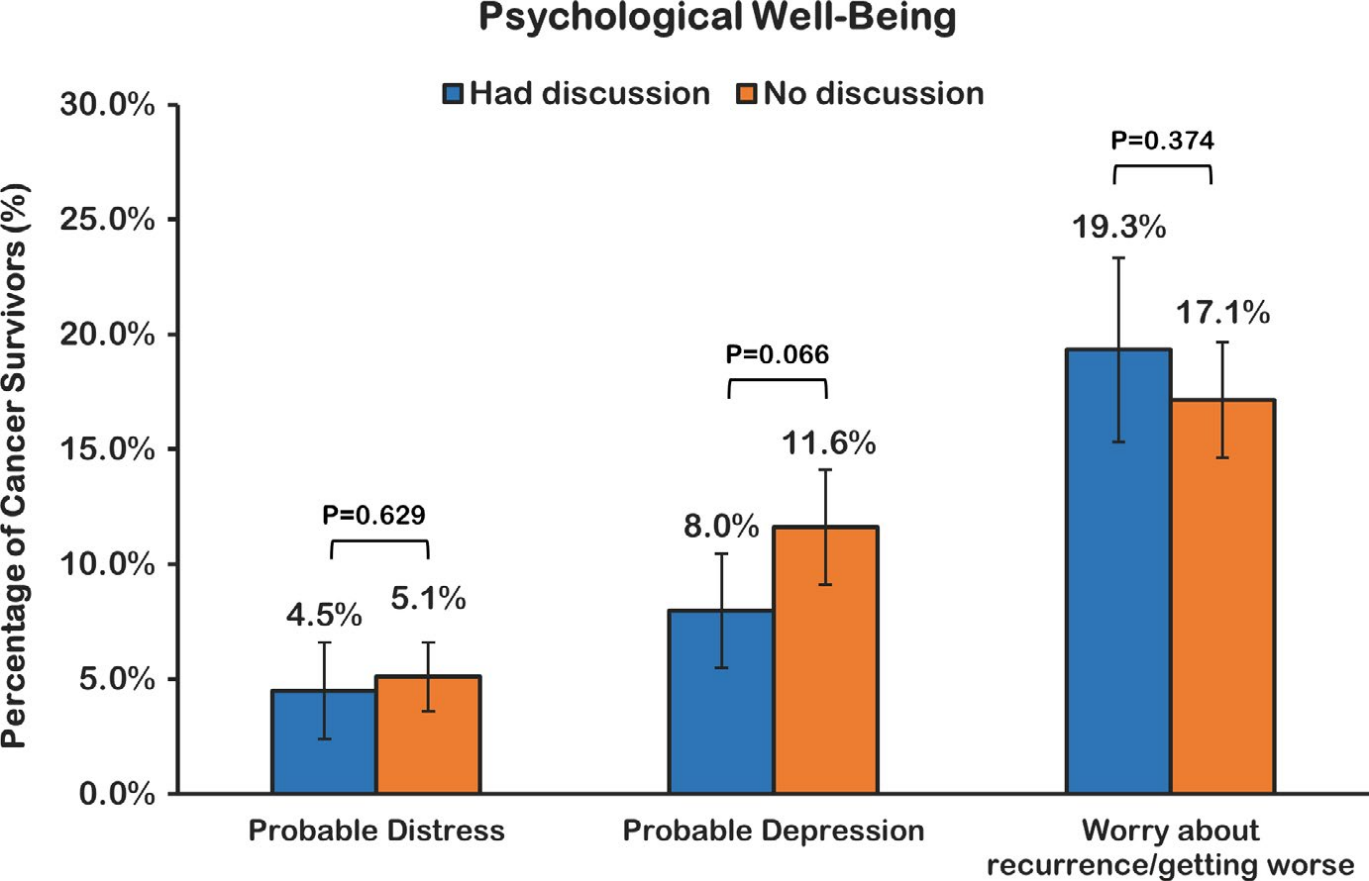
Unadjusted prevalence of psychological well‐being outcomes with cancer among U.S. Cancer survivors, by patient–provider discussion

The crude prevalence of psychological well‐being indicators is presented in Figure 1. We found no evidence of statistically significant differences between the discussion and non‐discussion groups. However, after adjusting for covariates, respondents in the discussion group had significantly lower odds of depressive symptoms (OR, 0.45; 95% CI, 0.26–0.77, *p* = 0.004; Table 2).

**TABLE 2:** Multivariable logistic regression results: psychological well‐being and attitude toward cancer care.

	PHQ2‐Depression Symptoms		K6‐Psychological Distress		Worry about cancer recurrence/getting worse		Positive Attitudes toward cancer care	
	Odds Ratio (95% CI)^a^	*p* value	Odds Ratio (95% CI)^a^	*p* value	Odds Ratio (95% CI)^a^	*p* value	Odds Ratio (95% CI)^a^	*p* value
Patient–provider discussion about emotional/social needs								
No	1.00 (Ref.)		1.00 (Ref.)		1.00 (Ref.)		1.00 (Ref.)	
Yes	0.45 (0.26–0.77)	0.004	0.60 (0.28–1.29)	0.1882	0.97 (0.68–1.38)	0.8571	1.97 (1.46–2.66)	<0.0001
Age group								
18–54	1.00 (Ref.)		1.00 (Ref.)		1.00 (Ref.)		1.00 (Ref.)	
55–64	0.54 (0.26–1.12)	0.0974	0.24 (0.10–0.60)	0.0024	0.43 (0.27–0.70)	0.0007	0.60 (0.38–0.94)	0.0258
65–74	0.27 (0.14–0.51)	<.0001	0.16 (0.07–0.37)	<.0001	0.25 (0.16–0.39)	<.0001	0.64 (0.39–1.04)	0.0729
75+	0.09 (0.04–0.19)	<.0001	0.04 (0.01–0.14)	<.0001	0.15 (0.09–0.26)	<.0001	0.52 (0.30–0.88)	0.0155
Sex								
Male	1.00 (Ref.)		1.00 (Ref.)		1.00 (Ref.)		1.00 (Ref.)	
Female	1.23 (0.78–1.95)	0.3686	1.28 (0.71–2.31)	0.4129	0.91 (0.65–1.28)	0.6021	1.73 (1.31–2.30)	0.0001
Race/ethnicity								
Non‐Hispanic White	1.00 (Ref.)		1.00 (Ref.)		1.00 (Ref.)		1.00 (Ref.)	
Non‐Hispanic Black	0.73 (0.41–1.29)	0.2722	0.40 (0.13–1.25)	0.1148	0.81 (0.46–1.44)	0.4802	3.45 (2.11–5.64)	<0.0001
Hispanic	1.27 (0.56–2.88)	0.5608	0.92 (0.44–1.90)	0.8117	1.49 (0.86–2.58)	0.1502	4.35 (2.71–6.97)	<0.0001
Other	1.62 (0.64–4.06)	0.3061	0.52 (0.17–1.61)	0.2525	1.44 (0.71–2.89)	0.3077	1.90 (1.02–3.56)	0.0436
Marital status								
Not married	1.00 (Ref.)		1.00 (Ref.)		1.00 (Ref.)		1.00 (Ref.)	
Married	0.69 (0.43–1.12)	0.1308	0.99 (0.40–2.41)	0.9738	0.99 (0.71–1.40)	0.986	1.14 (0.85–1.52)	0.3867
Education								
Less than high school graduate	1.00 (Ref.)		1.00 (Ref.)		11.00 (Ref.)		1.00 (Ref.)	
High school graduate	0.86 (0.50–1.48)	0.5793	0.61 (0.26–1.44)	0.2543	0.99 (0.62–1.59)	0.9733	0.92 (0.62–1.36)	0.6702
Some college or more	0.60 (0.32–1.13)	0.112	0.65 (0.22–1.93)	0.4334	1.10 (0.65–1.87)	0.7196	0.85 (0.55–1.31)	0.4456
Family income								
Low income (FPL <200%)	2.36 (1.25–4.46)	0.0085	3.31 (1.27–8.63)	0.0145	1.96 (1.23–3.12)	0.0048	0.86 (0.57–1.28)	0.4443
Middle income (FPL 200–400%)	1.34 (0.69–2.62)	0.3852	1.09 (0.39–3.06)	0.8665	1.70 (1.06–2.75)	0.0295	1.20 (0.82–1.75)	0.3392
High Income (FPL >400%)	1.00 (Ref.)		1.00 (Ref.)		1.00 (Ref.)		1.00 (Ref.)	
Employment								
Not employed	2.26 (1.23–4.14)	0.0086	11.44 (3.54–36.90)	<.0001	1.72 (1.14–2.61)	0.0129	0.64 (0.43–0.95)	0.0155
Employed	1.00 (Ref.)		1.00 (Ref.)		1.00 (Ref.)		1.00 (Ref.)	
Region								
Northeast	1.00 (Ref.)		1.00 (Ref.)		1.00 (Ref.)		1.00 (Ref.)	
Midwest	0.97 (0.47–2.00)	0.9326	0.30 (0.12–0.77)	0.0129	0.73 (0.43–1.24)	0.2484	0.78 (0.52–1.15)	0.203
South	0.75 (0.36–1.57)	0.4391	0.40 (0.17–0.92)	0.0322	0.74 (0.47–1.16)	0.1816	0.92 (0.63–1.34)	0.6521
West	0.73 (0.33–1.62)	0.4424	0.57 (0.24–1.37)	0.2071	0.79 (0.44–1.43)	0.433	0.84 (0.54–1.30)	0.4296
Current health insurance								
Private	1.00 (Ref.)		1.00 (Ref.)		1.00 (Ref.)		1.00 (Ref.)	
Public	1.14 (0.73–1.79)	0.5524	1.07 (0.47–2.45)	0.8691	1.05 (0.70–1.57)	0.8189	0.80 (0.59–1.08)	0.1437
Uninsured	0.97 (0.27–3.53)	0.9676	1.48 (0.29–7.48)	0.6336	2.77 (0.93–8.26)	0.0685	2.35 (0.83–6.66)	0.109
Number of chronic conditions								
0	1.00 (Ref.)		1.00 (Ref.)		1.00 (Ref.)		1.00 (Ref.)	
1	7.80 (3.01–20.25)	<.0001	2.70 (0.86–8.52)	0.0893	1.36 (0.76–2.43)	0.2969	1.01 (0.66–1.55)	0.9507
2	12.86 (5.33–31.04)	<.0001	7.23 (2.35–22.24)	0.0006	1.78 (1.02–3.10)	0.0423	1.05 (0.66–1.68)	0.8296
3+	20.61 (9.15–46.42)	<.0001	9.40 (3.25–27.17)	<.0001	1.52 (0.87–2.67)	0.142	0.88 (0.57–1.34)	0.5448
Time since last cancer treatment								
On treatment	1.00 (Ref.)		1.00 (Ref.)		1.00 (Ref.)		1.00 (Ref.)	
Post‐treatment								
1 to <5 years ago	0.90 (0.51–1.60)	0.7199	0.97 (0.45–2.10)	0.9333	0.83 (0.52–1.32)	0.4257	1.18 (0.82–1.69)	0.3666
5 to <10 years ago	1.17 (0.61–2.21)	0.6393	0.62 (0.28–1.39)	0.2417	0.29 (0.17–0.51)	<.0001	0.93 (0.63–1.39)	0.7363
10+ years ago	0.89 (0.53–1.51)	0.6644	0.83 (0.43–1.61)	0.5754	0.25 (0.16–0.40)	<.0001	1.24 (0.87–1.77)	0.2295
Cancer site^b^								
Breast	1.00 (Ref.)		1.00 (Ref.)		1.00 (Ref.)		1.00 (Ref.)	
Prostate	0.94 (0.47–1.85)	0.8458	1.21 (0.41–3.56)	0.724	0.95 (0.46–1.96)	0.8799	0.42 (0.25–0.71)	0.0014
Melanoma	0.27 (0.08–0.97)	0.0448	0.33 (0.05–2.02)	0.2276	0.75 (0.32–1.77)	0.5163	0.52 (0.29–0.92)	0.024
Colon	2.08 (0.79–5.45)	0.1376	1.20 (0.36–3.96)	0.7701	1.70 (0.92–3.16)	0.0913	0.61 (0.33–1.14)	0.1188
Cervical	1.64 (0.71–3.83)	0.2488	2.48 (0.84–7.32)	0.1002	0.77 (0.32–1.87)	0.5607	0.50 (0.24–1.06)	0.0689
Uterus	0.71 (0.19–2.68)	0.6107	0.07 (0.01–0.70)	0.024	0.47 (0.15–1.41)	0.1748	0.58 (0.30–1.10)	0.0949
Lung	0.72 (0.25–2.08)	0.5463	0.96 (0.18–5.12)	0.961	2.27 (1.10–4.68)	0.026	0.63 (0.29–1.36)	0.2383
Lymphoma	3.30 (1.15–9.50)	0.0267	1.64 (0.38–7.18)	0.5096	2.44 (0.96–6.19)	0.0601	0.46 (0.18–1.18)	0.107
Bladder	0.79 (0.13–4.60)	0.7886	1.38 (0.12–15.98)	0.795	1.28 (0.50–3.32)	0.6053	0.44 (0.22–0.91)	0.0268
Other	1.37 (0.72–2.60)	0.3398	1.31 (0.49–3.46)	0.5889	1.71 (1.05–2.79)	0.0318	0.53 (0.34–0.83)	0.0059
Multiple sites	0.99 (0.38–2.56)	0.9751	1.85 (0.61–5.63)	0.2804	2.32 (1.21–4.45)	0.0117	0.60 (0.35–1.02)	0.0611

Abbreviation: Ref, reference; FPL, federal poverty level.

^a^ Adjusted for age, sex, race/ethnicity, marital status, education, family income, employment, census region, health insurance, number of conditions other than cancer, and time since cancer treatment.

^b^ Estimated from a separate multivariable model including the same set of covariates above except sex.

### 3.3 Benefit findings

**FIGURE 2:**
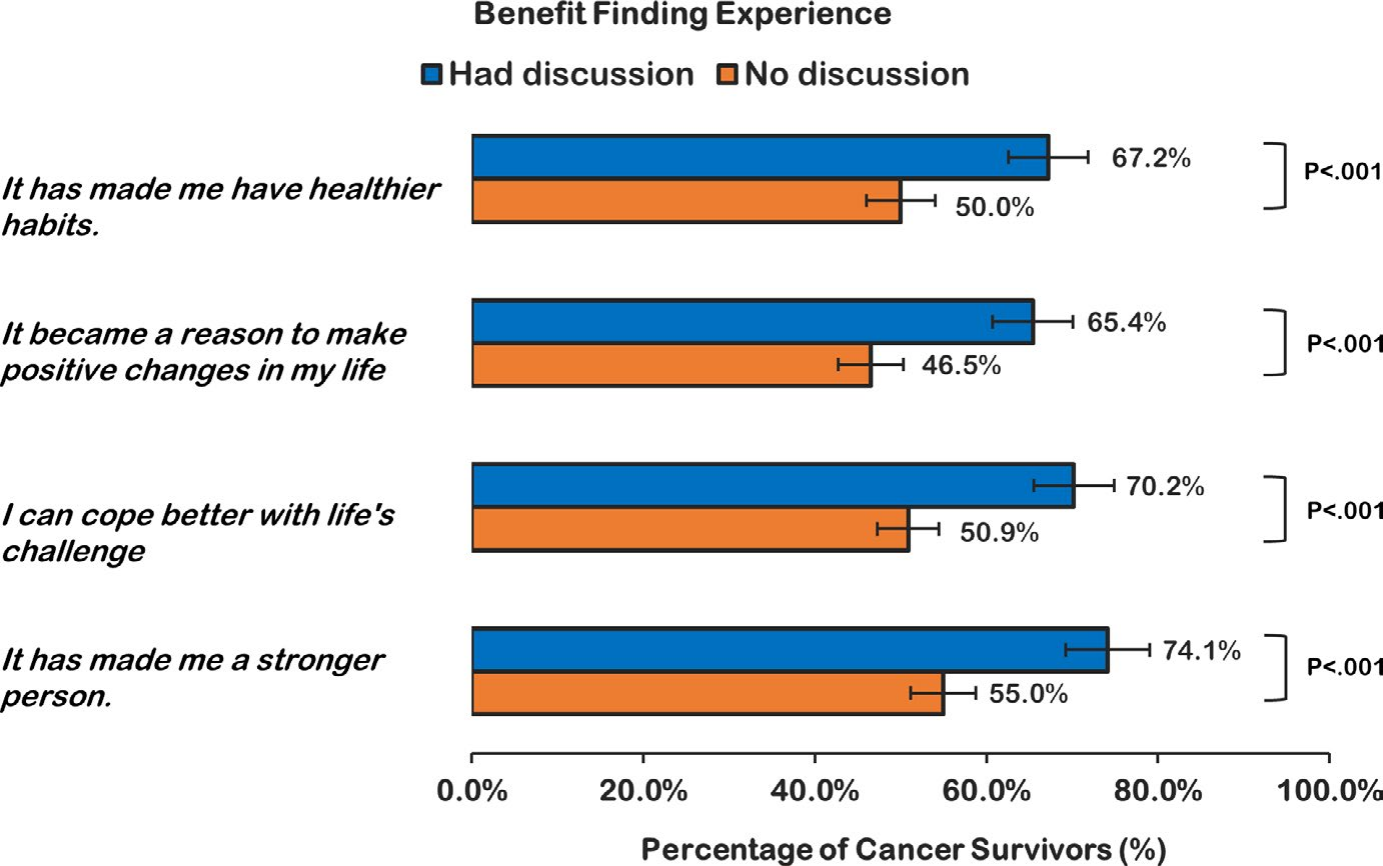
Unadjusted prevalence of benefit finding among U.S. Cancer survivors, by patient–provider discussion

Responses to questions on benefit finding experience after a cancer diagnosis are summarized in Figure 2, and crude prevalence of benefit findings was significantly higher in the group that reported patient–provider discussion. In the multivariable analyses, respondents in the discussion group had significantly higher odds of reportedly experiencing benefit finding (OR, 1.97; 95% CI, 1.46–2.66, *p* < 0.0001; Table 2).

### 3.4 Sensitivity analyses

Analyses using alternative definitions of patient–provider discussion did not appear to affect the main findings significantly (sensitivity analyses 1–3), with the exception that including only those having brief discussions did not have a significant marginal effect on depressive symptoms (sensitivity analysis 2). When including both brief and detailed discussions, we observed a statistically significant decrease in the likelihood of having severe psychological distress. Regardless of discussion quality (detailed vs. brief), patient–provider discussion about emotional and social needs was associated with experiencing benefit finding after a cancer diagnosis (sensitivity analyses 1–3). The main results also appeared insensitive to the inclusion of the non‐melanoma or unknown skin cancer patients (sensitivity analysis 4). The effect of patient–provider discussion was no longer significant when including those on active treatment only (sensitivity analysis 5) and become more potent when including those after treatment (sensitivity analysis 6).

## 4 DISCUSSION

Using a nationally representative survey, we examined the association between having a detailed patient–provider discussion about emotional and social needs and indicators of psychological well‐being among cancer survivors. Our results demonstrate that cancer survivors who had detailed discussion about emotional and social needs with any healthcare providers were less likely to have depressive symptoms and more likely to have a positive change in their attitude towards living with cancer. In a sensitivity analysis, we also observed that the effect of having any patient–provider discussion on severe psychological distress became significant. These findings suggest that patient–provider discussions about the emotional and social needs were associated with positive mental health outcomes and outlook on living with cancer.

Our findings represent a significant contribution to the literature because they address an important component of cancer care that involves attending to patients’ emotional and social needs, both of which are critical to achieving improved health outcomes.^5^, ^6^, ^42^, ^43^ There has been increased attention to patient–provider discussions about patients’ psychosocial needs, specifically as it is related to improved mental health outcomes.^28^, ^42^, ^44^ Prior evidence has made it apparent that diagnosis of cancer can have a substantial emotional impact on patients.^1^, ^2^, ^3^, ^4^ According to the mandate issued by the Institute of Medicine, survivorship care planning must take into account the psychosocial needs of patients.^27^ To our knowledge, this study is the first to quantitatively examine the association of patient–provider discussion about cancer survivors’ psychosocial needs with their mental health outcomes. Our findings are especially noteworthy, given that we found a significant association of patient–provider discussion about emotional and social needs with patients’ psychological well‐being and attitude towards cancer survivorship. Although there was no significant association between the patient–provider discussion and patients’ fear of cancer recurrence, a quality patient–provider discussion can increase the degree of social support and improve cancer survivors’ mental health‐related quality of life.^24^ Previous studies also showed that psychosocial care through patient–provider communication during cancer treatment and follow‐up was associated with improved HRQoL of localized prostate cancer survivors.^25^ Patients’ perceived risk of recurrence can be different from or greater than the actual risk the providers assess, which may not be captured and addressed adequately.^45^ Future studies should examine risk perception and fear of cancer recurrence among cancer survivors and further investigate what type of emotional support could be effective.

In this study, we also found that only about one‐third (33.6%) of cancer survivors reported having a detailed discussion with any healthcare provider about their emotional and social needs, which is concerning. This finding is supported by Pollak et al., showing that on average, oncologists responded only 22% of the time to patients’ emotional needs.^46^ Similarly, another study of colorectal cancer survivors found that little attention was directed towards the patients’ emotional and social needs.^47^ Although we were not able to distinguish provider type in this study, it has been reported that some providers in primary care or oncology may not be capable of addressing the patients’ psychosocial needs. For example, studies suggest that providers’ lack of clearly defined responsibilities in providing psychosocial care to the cancer survivors and reluctance to inquire about patients’ concerns and feelings are the reasons.^48^, ^49^ Other reasons, such as failing to address emotional health during cancer treatment from time constraints, patients’ concern of overburdening the providers with their emotional issues,^46^, ^50^ and the misconception that mental problems are unavoidable in cancer survivorship, were suggested in the previous studies.^51^, ^52^ Moreover, oncologists believe that dealing with the psychosocial problems of cancer patients is one of their toughest communication challenges.^53^ Previous analyses of cancer survivorship care found that most (61%–71%) of patient–provider discussions covered information on treatment or follow‐up care with little emphasis (26%–36% of the discussions) on patients’ emotional and social needs.^7^, ^8^, ^36^ To support primary care providers, in particular, healthcare systems could implement and evaluate routine patient‐reported outcome monitoring programs that assess factors such as depression symptoms and provide tools for providers to discuss depression with patients (e.g., training, clinical decision support).^54^, ^55^

Our study also found that cancer survivors with multiple chronic conditions are disproportionately affected by depressive symptoms, even after controlling for patient–provider discussions about psychosocial issues. Having a chronic condition, such as diabetes, requires extensive self‐management, similar to cancer. It is possible that as the number of chronic conditions increases, patients’ risk for depression increases due to the stressors associated with disease management.^48^, ^56^ Moreover, those with multiple conditions exhibit a significant decline in physical functioning, limiting interpersonal and social activities.^57^ These findings suggest that discussing the patients’ emotional and social needs may not be enough for cancer survivors suffering from multiple chronic conditions as it requires a greater degree of supportive communication from different specialty providers.^58^, ^59^ Despite an increase in cancer patients with contemporary depression symptoms,^3^, ^4^ many cancer patients (20%–40%) with depression or distress were not referred to psychosocial health services^60^, ^61^; even referred, approximately 20% of the patients never received psycho‐oncology counseling.^61^ Establishing the evidence of patient–provider communication could help steer efforts to improve the psychosocial aspect of patient–provider relations and adequate provision of psychosocial services.^27^, ^28^, ^29^ More research is needed to understand why cancer survivors with multiple chronic conditions are disproportionately affected by depression and what interventions may be helpful for addressing depression in this population.

### 4.1 Limitations

There are several limitations to our study. First, the cross‐sectional nature of our study precludes the directionality of the association between having patient–provider discussion and mental health outcomes. The MEPS‐ECSS is also limited by the data to make a distinction between whether the patient or the provider initiated the discussion about emotional and social needs, which may be subject to self‐selection bias; a patient who initiates discussion may more likely be experiencing mental health issues, and those who benefited from the discussion are more likely to remember it. Second, the item that we used for patient–provider discussion did not distinguish whether survivors had such discussion with the oncologist or other care providers (e.g., nurse, administrative staff, or social workers). Future studies should include the type of provider and further examine potential variations in the quality of patient–provider discussion and its associated patient health outcomes. Third, the information used in this study is self‐reported and could be subject to recall bias and social desirability bias. Last, this study is limited only to patient characteristics due to the lack of information about the providers, such as patient–provider relationship duration, which can be an important element instituting trust and rapport between the patient and provider.

### 4.2 Conclusions

Our findings from a representative population of cancer survivors in the United States illustrate the significance of provider engagement and consideration for cancer survivors’ emotional and social needs. Detailed patient–provider discussion of the patients’ emotional and social needs was associated with a lower likelihood of depressive symptoms and a higher likelihood of benefit findings in living with cancer. The prevalence of patients reporting a detailed discussion with their provider was low—only 33.6% in our study—highlighting the need to identify and overcome barriers to the patient–provider discussion about psychosocial needs in cancer survivorship care delivery for improved mental health outcomes.

## CONFLICT OF INTEREST

The views expressed here are those of the authors and not their affiliated institutions. All authors have nothing to disclose.

## ETHICAL APPROVAL

This study was reviewed by the University of Florida Institutional Review Board. No ethical approval was needed because it uses data that are completely deidentified and available to the public.

## Data Availability

The data that support the findings of this study are available from the Agency for Healthcare Research and Quality at https://meps.ahrq.gov/mepsweb/
